# Effect of standard‐dose and high‐dose pimobendan on select indices of renal and cardiac function in dogs with American College of Veterinary Internal Medicine stage B2 myxomatous mitral valve disease

**DOI:** 10.1111/jvim.16537

**Published:** 2022-09-13

**Authors:** Joanna L. Kaplan, Lance C. Visser, Catherine T. Gunther‐Harrington, Eric S. Ontiveros, Luke A. Wittenburg, Carrie A. Palm, Joshua A. Stern

**Affiliations:** ^1^ Department of Medicine & Epidemiology, School of Veterinary Medicine University of California Davis California USA; ^2^ Department of Surgical & Radiological Sciences, School of Veterinary Medicine University of California Davis California USA

**Keywords:** azotemia, canine, degenerative, kidney function, subclinical

## Abstract

**Background:**

Pimobendan might have favorable effects on renal function but this has not been well‐studied in dogs with myxomatous mitral valve disease (MMVD).

**Objectives:**

Determine the effects of standard‐dose (SD_pimo) and high‐dose pimobendan (HD_pimo) on glomerular filtration rate (GFR) and cardiac size and function in dogs with preclinical MMVD.

**Animals:**

Thirty nonazotemic dogs with stage B2 MMVD.

**Methods:**

Prospective, randomized, double‐blinded, placebo‐controlled clinical study. Dogs had an echocardiographic examination, assessment of GFR (iohexol clearance), N‐terminal probrain natriuretic peptide (NT‐proBNP), and quality of life (QOL) score at baseline and 7 to 10 days after placebo (n = 6), SD_pimo 0.2 to 0.3 mg/kg q12 (n = 12), or HD_pimo 0.5 to 0.6 mg/kg q12h (n = 12).

**Results:**

No significant differences in GFR or QOL scores were detected between groups (*P* ≥.07). After HD_pimo, the mean [SD] percent change of NT_proBNP (−46.1 [20.2]%), left atrial volume (LAV; −27.1 [16.9]%), left ventricular end‐diastolic volume (EDV; −21.8 [15.0]%), and end‐systolic volume (ESV; −55.0 [20.7]%) were significantly different (*P* ≤.004) from placebo (0.5 [19.9]%, 1.3 [15.6]%, −0.2 [8.2]%, −7.3 [35.6]%, respectively) but not the percent change after SD_pimo (−36.6 [16.1]%, −22.7 [14.9]%, −16.7 [12.5]%, −41.6 [14.8]%, respectively; *P* > .05). After SD_pimo, percent change of NT_proBNP, LAV, EDV, and ESV were significantly different from placebo (*P* < .05).

**Conclusions and Clinical Importance:**

Results suggest that pimobendan (SD_pimo or HD_pimo) might not affect renal function in nonazotemic dogs with stage B2 MMVD. High‐dose pimobendan did not demonstrate advantages over SD_pimo within the constraints of our study.

**Abbreviations**
ACVIM: American College of Veterinary Internal Medicine
BUN: blood urea nitrogen
CHF: congestive heart failure
EDV: end‐diastolic volume of the left ventricle
EF: ejection fraction of the left ventricle
ESV: end‐systolic volume of the left ventricle
FETCH: Functional Evaluation of Cardiac Health
FS: fractional shortening
GFR: glomerular filtration rate
HD_pimo: high‐dose pimobendan
MMVD: myxomatous mitral valve disease
MR: mitral valve regurgitation
NT‐proBNP: N‐terminal probrain natriuretic peptide
QOL: quality of life
SD_pimo: standard‐dose pimobendan
SDMA: symmetric dimethylarginine

## 1 INTRODUCTION

Myxomatous mitral valve disease (MMVD) is the most common cardiovascular disease and the leading cause of congestive heart failure (CHF) in dogs.^1^, ^2^ Renal dysfunction is a common finding in dogs with MMVD, particularly as the disease progresses.^3^, ^4^ Renal dysfunction might complicate clinical management and adversely impact quality of life and outcome in dogs with MMVD.^5^, ^6^ Therefore, it is important to understand how drugs used in the clinical management of MMVD affect renal function.

Pimobendan is an inodilator drug recommended for dogs with American College of Veterinary Internal Medicine (ACVIM) stage B2, C, and D MMVD.^1^ In contrast to other drugs used in the management of MMVD (vasodilators, diuretics),^7^, ^8^ pimobendan does not activate the renin angiotensin aldosterone system (RAAS).^9^, ^10^, ^11^ In preclinical studies of healthy dogs, pimobendan increases cardiac output,^12^ renal blood flow,^13^ and offsets furosemide‐induced prerenal azotemia.^11^ Pimobendan might have favorable effects on renal function and increase glomerular filtration rate (GFR), particularly at high doses.^11^ However, 1 study in healthy dogs^14^ and another in dogs with experimentally‐induced mitral valve regurgitation^13^ suggest that pimobendan does not have an important effect on GFR. Off‐label escalation of pimobendan dose, frequency, or both has become a clinical strategy for dogs with MMVD and refractory CHF, azotemia, or both.^1^, ^15^, ^16^ Although the benefits of standard‐dose pimobendan (SD_pimo) on cardiac function are well‐documented in dogs with MMVD,^17^, ^18^, ^19^ to our knowledge, the effect of SD_pimo and particularly high‐dose pimobendan (HD_pimo) on renal and cardiac function have not been well‐studied.

The primary objective of this study was to determine the effect of SD_pimo (0.2‐0.3 mg/kg PO q12) and HD_pimo (0.5‐0.6 mg/kg PO q12) on glomerular filtration rate (GFR) in dogs with ACVIM stage B2 MMVD. A secondary objective was to determine the effect of SD_pimo and HD_pimo on echocardiographic indices of cardiac size and function. We hypothesized that SD_pimo and HD_pimo would improve renal and cardiac function.

## 2 MATERIALS AND METHODS

All study procedures were approved by the Institutional Animal Care and Use Committee at the University of California, Davis (protocol #: 20226). All dog owners gave written, informed consent prior to enrollment.

### 2.1 Animals

Study subjects were client‐owned dogs referred to the University of California, Davis Veterinary Medical Teaching Hospital either for routine evaluation of suspected cardiovascular disease or for the purposes of this study. Dogs eligible for inclusion had to be ≥6 years of age, have a body weight of ≥2.5 kg and ≤20 kg, have a characteristic left apical systolic murmur grade ≥3 of 6, and had to be free of clinical signs of CHF. Dogs were required to have echocardiographic evidence of MMVD defined as characteristic valvular lesions including valve thickening/irregularity, leaflet prolapse, or both, and systolic mitral valve regurgitation (MR) based on color Doppler, and fulfill the echocardiographic left atrial and ventricular heart size criteria for ACVIM Stage B2 MMVD.^1^ Specifically, dogs with a short‐axis left atrium to aortic root ratio (LA/Ao_Sx) ≥1.6 and a left ventricular internal dimension at end‐diastole (LVIDd) normalized to body weight (LVIDdN) ≥1.7 cm/kg^0.294^. Vertebral heart score >10.5 was not verified. Dogs could not have any additional cardiac disease, including suspected concurrent precapillary pulmonary hypertension with a tricuspid regurgitation velocity >4 m/s. Dogs also had to be free of radiographic evidence of cardiogenic pulmonary edema (determined by a cardiology resident and board‐certified cardiologist). Dogs were not permitted to be receiving medications known to affect the cardiovascular system or renal function within 14‐days of enrollment. Dogs could not have a known or preexisting systemic or organ‐specific disease, including azotemia defined by a serum creatinine >1.5 mg/dL, our hospital's upper reference limit. Dogs were also excluded if they had an uncooperative temperament that required sedation for any study procedure, clinically important brady‐ or tachyarrhythmias defined as those requiring antiarrhythmic therapy by the attending cardiologist, or systemic hypertension defined as a systolic arterial blood pressure >180 mm Hg by Doppler sphygmomanometry.

### 2.2 Study design

This was a single‐site, prospective, randomized, double‐blinded, placebo‐controlled clinical study. Before inclusion (at the study screening visit), the case history (including diet and medications) was reviewed for each dog. Physical examination, systolic arterial blood pressure, echocardiographic examination, and thoracic radiography were performed. The screening echocardiographic examination was reviewed by 2 primary study investigators (JLK, LCV). Packed cell volume with total solids, a serum biochemistry with creatinine, blood urea nitrogen (BUN), electrolyte concentrations, and urine specific gravity were assessed. Once enrollment eligibility was confirmed, owners were scheduled to drop off their dogs within 5‐days of the study screening visit. Owners were instructed to not alter their dog's diet throughout the study period and to fast their dogs 12‐hours prior to each study visit. Free access to water was permitted.

At baseline and 7 to 10 days after, each dog underwent a physical examination, an echocardiographic examination, systolic arterial blood pressure assessment, and phlebotomy for assessment of packed cell volume and total solids, serum creatinine, BUN, electrolytes, and symmetric dimethylarginine (SDMA), plasma N‐terminal probrain natriuretic peptide (NT‐proBNP), and GFR by plasma clearance of iohexol (Omnipaque 300 GE healthcare, Chicago, Illinois) was performed. Dog owners were instructed to fill out a previously validated Functional Evaluation of Cardiac Health (FETCH) questionnaire where higher scores are suggestive of worse quality of life in dogs with cardiac disease.^20^

Dogs were randomly allocated to receive SD_pimo 0.2 to 0.3 mg/kg PO q12, HD_pimo 0.5 to 0.6 mg/kg PO q12 (Vetmedin 1.25 mg, 2.5 mg, 5 mg, and 10 mg chewable tablets, Boehringer Ingelheim Vetmedica, Inc, Duluth, Georgia) or placebo (Cosequin DS chewable tablet, Nutramax Laboratories, Inc, Lancaster, South Carolina). Randomization, drug preparation, and distribution were carried out by an independent veterinary pharmacist and technician not involved in the study and unaware of any patient information other than group assignment. Each treatment group (SD_pimo and HD_pimo) used a 2:1 allocation ratio relative to placebo. Thus, each treatment group had twice as many dogs as placebo group. The placebo group was primarily included to account for day‐to‐day variability of the echocardiographic and renal function variables. Study investigators and owners were blinded to the group assignment. Each owner was instructed to administer the final treatment <1 hour prior to the final study visit, which was verified verbally. All diagnostics including the echocardiographic examination and phlebotomy were performed 1 to 3 hours posttreatment.

### 2.3 Blood sample collection and analysis

All blood samples were collected by jugular venipuncture. Analysis of serum creatinine, BUN, electrolytes, as well as packed cell volume, and total solids was performed and analyzed immediately through our in‐house diagnostic laboratory. An attempt was made to collect urine (free catch or cystocentesis) from all dogs and, if collected, urine specific gravity was determined. Samples for SDMA were sent to IDEXX for immediate analysis. Blood samples for iohexol concentrations and GFR analysis, as well as NT‐pro‐BNP concentrations were collected in lithium heparin tubes and ethylenediaminetetraacetic acid (EDTA) tubes, respectively. These were centrifuged at 2000*g* for 15 minutes within 20 minutes of collection, and plasma was separated, aliquoted into cryotubes containing 300 μL of each sample, and stored in at −80°C for future analysis.^21^ Samples for NT‐proBNP were sent in batch to IDEXX laboratories for analysis once all samples were obtained.

### 2.4 Glomerular filtration rate measurements and calculations

After placement of an intravenous cephalic catheter, an intravenous injection of iohexol (Omnipaque concentration of 300 mg I/mL) at a dose of 1 mL/kg was administered as a bolus. Blood samples (2 mL) for iohexol concentration were collected at 2‐, 3‐, and 4‐hours after iohexol administration. All blood samples for GFR analysis were collected in lithium heparin tubes. Once all samples were obtained, plasma iohexol concentrations were measured in batch using a high‐performance liquid chromatography‐tandem mass spectrometry (LC‐MS/MS) method designed and validated by the Developmental Cancer Therapeutics Laboratory (Department of Surgical and Radiological Sciences, UC Davis School of Veterinary Medicine).^22^, ^23^ Briefly, 45 μL of lithium heparin plasma was precipitated with 500 μL acetonitrile containing internal standard (iothalamate) and vortex mixed. Following centrifugation at 20 000*g* for 10 minutes, 10 μL of supernatant was added to 990 μL 5% acetonitrile containing 0.1% formic acid for injection onto the HPLC system. Concentrations were calculated by linear regression of iothalamate (*m/z* 614.4 → 233.1 amu) to iohexol (sum *m/z* 822.1 → 803.8/602.9/656.6 amu) peak areas using a calibration curve generated in fortified canine lithium heparin plasma made of 8 nonzero concentrations ranging from 25 to 1000 μg/mL. Four sets of quality control samples at 50, 200 and 600 μg/mL were made and analyzed with the dog samples. The calibration curve was linear from 25 to 1000 μg/mL with accuracies greater than 90% at all concentrations. The accuracy and precision of the quality control samples were both within 7% at all concentrations. Iohexol plasma clearance rate (mL/min) for each subject was determined by using the 3‐sample plasma clearance method and calculating the dose/area under the curve. Area under the curve was defined as the area under the plasma concentration versus time curve from time 0 to 4 hours using a noncompartmental model^24^, ^25^, ^26^, ^27^, ^28^, ^29^, ^30^, ^31^, ^32^, ^33^ generated with the use of commercially available software (Phoenix WinNonlin, Version 8.3, Certara Inc, Princeton, New Jersey). Clearance values were then normalized to body weight (mL/min/kg). For the purposes of this study, GFR values >2 mL/kg/min were considered normal (International Renal Interest Society, http://www.iris-kidney.com/education/gfr.html).^3^

### 2.5 Echocardiographic examinations

#### 2.5.1 Image acquisition

All echocardiographic examinations were performed by 2 study investigators, a cardiology resident (JLK) and a board‐certified cardiologist (LCV). Baseline and follow‐up echocardiograms for each dog were performed by the same study investigator and using the same ultrasound unit (Philips EPIQ 7C, Philips Healthcare, Andover, MA) to maintain consistency in image acquisition. Standard tomographic imaging planes were utilized.^34^ Dogs were gently restrained in right and left lateral recumbency with a simultaneous ECG, and at least 6 cardiac cycles were acquired from each imaging plane.

#### 2.5.2 Echocardiographic measurements

All echocardiographic measurements were performed at a digital off‐cart workstation (Syngo Dynamic Workplace, Siemens Medical Solutions, Inc, Malvern, Pennsylvania) by a single investigator (JLK) en bloc after all dogs completed the study. This investigator was blinded to the dog's clinical information, drug status, and date of echocardiogram. The final value recorded for each measurement was based on the average of 3 consecutive cardiac cycles.

From the right parasternal long‐axis 4‐chamber view optimized for the left atrium, maximum left atrial volume (LAV) was measured at ventricular end‐systole (immediately prior to mitral valve opening) by manually tracing the internal border of the LA and applying monoplane Simpson's method of discs.^35^ Left ventricular volume was estimated by manually tracing the left ventricular (LV) internal border and applying monoplane Simpson's method of discs from a right parasternal long‐axis 4‐chamber view optimized for the elongated LV at end‐diastole (EDV) and end‐systole (ESV).^35^ From the right parasternal short‐axis high papillary muscle view, left ventricular internal dimension at end‐diastole (LVIDd) and end‐systole (LVIDs) were measured from a 2D‐guided M‐mode where the cursor transected the midpoint of the septal arc through the LV free wall, equidistant from the 2 papillary muscles. These were measured using the leading edge to leading edge technique. End‐diastole was timed to the onset of the R wave on the ECG and end‐systole represented the minimum chamber dimension. The LA/Ao_Sx was measured from the right parasternal short‐axis basilar view optimized for visualization of the aortic valve cusps at the level of the aortic root and LA.^36^, ^37^ Measurements were performed using an inner edge to inner edge technique upon visualization of aortic valve closure (early diastole). The diameter of the aortic root was measured from the midpoint of the convex curvature of the internal wall of the right aortic sinus of Valsalva and continuing along the commissure of the left and noncoronary cusps to the junction of the aortic wall, left coronary cusp, and noncoronary cusp. The LA measurement extended along this trajectory starting from the internal border of the LA to the internal border of the far‐field LA wall. Care was taken to avoid extending the measurement to within a pulmonary vein.

The LVIDd measurement was normalized to body size as follows: LVIDd (cm)/body weight (kg)^0.294^.^1^, ^38^ All cardiac chamber volume estimates were indexed to (ie, divided by) body weight (kg). Fractional shortening (FS) was calculated as ([LVIDd − LVIDs]/LVIDd) × 100. Ejection fraction (EF) was calculated as ([EDV − ESV]/EDV) × 100.

### 2.6 Statistical analysis

Statistical analyses were performed using commercial computer software (MedCalc Statistical Software, MedCalc Software bvba, Ostend, Belgium). Sample size of 12 dogs per pimobendan treatment group was based on a 2‐tailed, paired samples *t*‐test model using a statistical power of 0.8, an estimated effect size of 20% change in GFR, the primary outcome variable in this study, and type I error set to 0.05. Several resources were consulted to help estimate the desired effect size of GFR including studies evaluating day‐to‐day variability of GFR,^32^, ^39^, ^40^ a previous study assessing GFR in healthy dogs before and after pimobendan,^14^ and a study evaluating GFR in dogs with MMVD.^3^ Normality testing was performed using the D'Agostino‐Pearson test. Baseline variables were compared using a 1‐way ANOVA and Tukey‐Kramer post hoc test (parametric data) or a Kruskal‐Wallis and Conover post hoc test (nonparametric data). Before and after treatment comparisons were made with a paired *t* test or Wilcoxon test. Where statistically significant within group differences were identified, percent changes were calculated for each individual dog and compared among groups with an ANOVA and Tukey‐Kramer post hoc test (or nonparametric equivalent). Statistical significance was set at *P* < .05.

## 3 RESULTS

Thirty‐one dogs with ACVIM stage B2 MMVD were enrolled in this study. One dog was withdrawn from the study at its owner's request due to perceived behavior changes of aggression toward the owner and ataxia after receiving several doses of the study medication (later determined to be HD_pimo). Thus, 30 dogs completed the study. Twelve dogs were enrolled in each pimobendan group and 6 were enrolled in the placebo group. Breeds enrolled in the SD_pimo group consisted of 8 mixed breed dogs, 2 Chihuahuas, 1 Pomeranian and 1 Australian Shepherd. Dogs in the HD_pimo group consisted of 3 mixed breed dogs, 3 Cavalier King Charles Spaniels, and 1 each of the following: Pekingese, Schipperke, Cairn terrier, Dachshund, Boston terrier and Yorkshire terrier. The placebo group consisted of 2 mixed breed dogs and 1 each of the following: Cavalier King Charles Spaniel, Chihuahua, Jack Russel terrier and Yorkshire terrier.

Study population characteristics are summarized in Table 1. There were no statistically significant differences in clinical, clinicopathological, or echocardiographic variables, or owner perceived quality of life (FETCH) scores among the groups, aside from dogs enrolled in the HD_pimo group weighed slightly but significantly more (*P* = .05) than dogs enrolled in the SD_pimo group. One dog in the placebo group, and 3 each in the SD_pimo and HD_pimo groups had a baseline GFR <2 mL/kg/min.

**TABLE 1:** Baseline study sample demographics of dogs with ACVIM stage B2 myxomatous mitral valve disease

Baseline variables	Placebo (n = 6)	SD_Pimo (n = 12)	HD_Pimo (n = 12)	*P*‐value
Clinical and laboratory variables				
Age (years)	10.2 (1.4)	11.1 (2.6)	10.0 (1.7)	.46
Bodyweight	7.1 (1.4)	6.5 (3.1)	9.6 (3.4)^a^	**.05**
BP (mm Hg)	145 (13)	144 (21)	138 (16)	.65
PCV (%)	47 (8)	51 (5)	48 (4)	.25
SDMA (μg/dL)	9.0 (1.7)	9.0 (2.2)	10.0 (3.2)	.62
Creatinine (mg/dL)	0.8 (0.1)	0.9 (0.3)	0.8 (0.2)	.69
BUN (mg/dL)	20.0 (5.1)	21.4 (7.3)	16.3 (2.8)	.08
GFR (mL/min/kg)	2.8 (2.5, 3.5)	2.8 (2.0, 3.7)	2.3 (2.0, 3.2)	.71
NT‐proBNP (pmol/L)	1174 (1010, 1672)	1228 (609, 2441)	1030 (678, 2685)	.99
FETCH score	2.0 (0.0, 9.0)	1.0 (0.0, 7.5)	4.5 (1.3, 6.0)	.78
Echocardiographic variables				
LA/Ao_Sx	2.1 (0.6)	2.0 (0.1)	2.0 (0.3)	.93
LVIDdN (cm/kg^0.294^)	1.9 (0.3)	1.8 (0.2)	1.9 (0.2)	.66
FS (%)	50.6 (3.5)	48.8 (9.1)	45.7 (6.0)	.36
LAV (mL/kg)	3.0 (1.0)	2.8 (0.8)	3.3 (1.4)	.51
EDV (mL/kg)	3.6 (3.3, 4.0)	3.7 (3.4, 3.8)	3.6 (3.1, 4.4)	.88
ESV (mL/kg)	0.9 (0.3)	1.0 (0.4)	1.1 (0.3)	.53
EF (%)	78.5 (4.3)	74.4 (7.2)	71.3 (8.3)	.16

*Note*: Data reported as mean (SD) or median (25th percentile, 75th percentile). *P*‐values that appear in bold denote statistical significance.

Abbreviations: BP, blood pressure; BUN, blood urea nitrogen; EDV, end‐diastolic volume of the left ventricle; EF, ejection fraction of the left ventricle; ESV, end‐systolic volume of the left ventricle; FETCH, functional evaluation of cardiac health; FS, fractional shortening; GFR, glomerular filtration rate; HD_pimo, high‐dose pimobendan; LA/Ao_Sx, left atrium to aortic root ratio in short‐axis; LAV, left atrial volume; LVIDdN, left ventricular internal dimension at end‐diastole normalized to body weight; NT‐proBNP, N‐terminal probrain natriuretic peptide; SD_pimo, standard‐dose pimobendan; SDMA, symmetric dimethylarginine.

^a^ Significantly different (*P* < .05) from SD_Pimo group.

Results of GFR, NT‐proBNP, FETCH score and echocardiographic indices of cardiac size and function before and 7 to 10 days after treatment are summarized in Table 2. No statistically significant differences in GFR or FETCH scores were identified within the SD_pimo or HD_pimo groups. However, NT‐proBNP and echocardiographic indices of cardiac size (LAV and EDV) exhibited statistically significant decreases relative to baseline within the SD_pimo and HD_pimo groups. Echocardiographic indices of systolic function (ESV and EF) were significantly changed (decreased for ESV and increased for EF) relative to baseline within the SD_pimo and HD_pimo groups. Regarding the among group comparisons in percent change in NT‐proBNP, LAV, EDV, and ESV, and EF, there were no statistically significant differences in the percent change after standard‐dose pimobendan compared to after high‐dose pimobendan. When percent changes were compared among groups, the only statistically significant differences identified were between the placebo group and groups receiving pimobendan.

**TABLE 2:** Mean (SD) or median (25th percentile, 75th percentile) of clinical, clinicopathological, and echocardiographic variables before and 7 to 10 days after placebo, standard‐dose pimobendan (SD_pimo, 0.2‐0.3 mg/kg PO q12h), and high‐dose pimobendan (HD_pimo, 0.5‐0.6 mg/kg PO q12h) in dogs with ACVIM stage B2 myxomatous mitral valve disease

Lab variables	Group	Before	After	*P*‐value (within groups)	Percent change	*P*‐value (among groups)
PCV (%)	Placebo	47 (8)	45 (5)	.99	–	–
	SD_pimo	51 (5)	48.3 (8)	.09	–	
	HD_pimo	48 (4)	44.6	.07	–	
SDMA (μg/dL)	Placebo	9.0 (1.7)	8.8 (1.8)	.87	–	–
	SD_pimo	9.0 (2.2)	8.9 (1.9)	.79	–	
	HD_pimo	10.0 (3.2)	10.8 (3.1)	.68	–	
Creatinine (mg/dL)	Placebo	0.8 (0.1)	0.7 (0.1)	.36	–	–
	SD_pimo	0.9 (0.3)	0.8 (0.2)	.21	–	
	HD_pimo	0.8 (0.2)	0.8 (0.2)	14	–	
GFR (mL/min/kg)	Placebo	3.0 (1.5)	3.9 (1.2)	.3	–	–
	SD_pimo	2.8 (2.0, 3.7)	3.6 (3.0, 5.1)	.07	–	
	HD_pimo	2.7 (1.2)	2.8 (1.1)	.77	–	
NT‐proBNP (pmol/L)	Placebo	1663 (1474)	1587 (1306)	.55	0.5 (19.9)	**<.001**
	SD_pimo	1615 (1202)	959 (695)	**.002**	−36.6 (16.1)^a^	
	HD_pimo	1030 (678, 2685)	598 (292, 1632)	**.002**	−46.1 (20.2)^a^	
FETCH score	Placebo	2.0 (0.0, 9.0)	5.0 (0.8, 7.3)	.58	–	–
	SD Pimo	1.0 (0.0, 7.5)	2.5 (1.0, 3.0)	.6	–	
	HD Pimo	4.5 (1.3, 6.0)	3.0 (2.0, 8.5)	.44	–	
LAV (mL/kg)	Placebo	3.0 (1.0)	3.1 (1.2)	.68	1.3 (15.6)	**.004**
	SD_pimo	2.8 (0.8)	2.1 (0.6)	**.002**	−22.7 (14.9)^a^	
	HD_pimo	3.3 (1.4)	2.4 (1.1)	**<.001**	−27.1 (16.9)^a^	
EDV (mL/kg)	Placebo	4.0 (1.1)	3.9 (0.8)	.1	−0.2 (8.2)	**.009**
	SD_pimo	3.8 (0.7)	3.2 (0.7)	**<.001**	−16.7 (12.5)^a^	
	HD_pimo	3.7 (0.8)	2.9 (0.8)	**.001**	−21.8 (15.0)^a^	
ESV (mL/kg)	Placebo	0.9 (0.3)	0.8 (0.3)	.47	−7.3 (35.6)	**<.001**
	SD_pimo	1.0 (0.4)	0.6 (0.3)	**<.001**	−41.6 (14.8)^a^	
	HD_pimo	1.1 (0.3)	0.5 (0.3)	**<.001**	−55.0 (20.7)^a^	
EF (%)	Placebo	78.5 (4.3)	80.3 (6.7)	.49	2.3 (7.3)	**.007**
	SD_pimo	74.4 (7.2)	82.5 (5.4)	**<.001**	11.3 (7.8)	
	HD_pimo	71.3 (8.3)	83.5 (8.9)	**<.001**	17.6 (10.4)^a^	

*Note*: *P*‐values that appear in bold denote statistical significance.

Abbreviations: EDV, end‐diastolic volume of the left ventricle; EF, ejection fraction of the left ventricle; ESV, end‐systolic volume of the left ventricle; FETCH, functional evaluation of cardiac health; GFR, glomerular filtration rate; HD_pimo, high‐dose pimobendan; LAV, left atrial volume; NT‐proBNP, N‐terminal probrain natriuretic peptide; SD_pimo, standard‐dose pimobendan; SDMA, symmetric dimethylarginine.

^a^ Significantly different (*P* < .05) compared to percent change of the placebo group.

No adverse effects, adverse clinical events or clinical signs were reported in any of the dogs that completed the study. However, as previously mentioned, 1 dog that received HD_pimo withdrew from the study due to owner perceived aggressive behavior and ataxia. Hydration status based on physical examination and total solids remained static at each examination. One dog in the SD_pimo group developed a mild anemia, which was noted at the second study visit. The PCV decreased from 50% to 32% (hematocrit = 27.6%). This anemia was self‐limiting and resolved in 72‐hours, which was verified with a complete blood count at the dog's primary veterinarian.

## 4 DISCUSSION

Results of this study did not support our hypothesis that pimobendan increases GFR (as assessed by iohexol clearance) in nonazotemic dogs with ACVIM stage B2 MMVD. The study did document changes in cardiac chamber size, systolic function and NT‐proBNP secondary to both SD_pimo and HD_pimo that are presumed to be beneficial. However, HD_pimo did not demonstrate a further change relative to SD_pimo within the confines of our study.

These results suggest that SD_pimo and HD_pimo neither improve nor worsen GFR in the short‐term in dogs with ACVIM stage B2 MMVD. Our results corroborate previous studies evaluating the effect of SD_pimo on GFR in healthy dogs^14^ and dogs with experimentally‐induced MR,^13^ which also failed to identify statistically significant changes in GFR attributable to pimobendan. Thus, our study supports the hypothesis that any enhanced renal blood flow to due pimobendan's positive effect on cardiac output might be limited by renal arterial vasodilation, which was demonstrated in an invasive hemodynamic study of healthy anesthetized dogs.^12^ Despite documenting improved myocardial function, cardiac output, and increased blood flow to some organs (eg, liver), pimobendan did not seem to enhance GFR in the dogs studied to date.^12^

To our knowledge, the effects of HD_pimo on cardiac and renal function have not been well‐studied in dogs with cardiovascular disease. One study evaluated SD_pimo and HD_pimo in dogs with experimentally‐induced MR and found that pimobendan reduced LA pressure in a dose‐dependent manner.^16^ Another study found that HD_pimo did not suppress or potentiate furosemide‐induced renin angiotensin aldosterone system activation in healthy dogs.^11^ These studies have prompted support for increasing pimobendan dose, frequency (q8h administration), or both for dogs with refractory CHF.^1^, ^15^ Additionally, some clinicians prescribe HD_pimo for the potential added benefit that pimobendan might improve renal function in the setting of furosemide‐induced prerenal azotemia,^11^ and because renal dysfunction is common in dogs with advanced stages of MMVD.^3^, ^4^ Our results do not support the broad notion that HD_pimo improves renal function, as measured by GFR. However, our results do suggest that HD_pimo is tolerated by most dogs, does not appear to worsen renal function, and has presumably favorable effects on echocardiographic indices of cardiac size and function and NT‐proBNP in nonazotemic dogs with ACVIM stage B2 MMVD.

One of 13 dogs that received HD_pimo developed clinical signs perceived by the owner to be ataxia and aggressive behavior that could have been secondary to HD_pimo. It is also possible these findings are coincidental as causation could not be confirmed. There is well established safety data for the recommended dose of pimobendan and, to our knowledge, no safety data for HD_pimo. It is possible that higher doses of pimobendan administered in this study might overlap with those where evidence of toxicity has been observed in previous studies.^41^, ^42^

This study failed to directly demonstrate a dose‐dependent effect of pimobendan on echocardiographic indices of cardiac chamber size and systolic function in the short‐term. This is in agreement with a previous study on dogs with experimentally‐induced MR.^16^ This study^16^ did report positive dose‐dependent hemodynamic effects of pimobendan on lowering left atrial pressure, increasing cardiac output, and reduced MR severity (based on jet area ratio), despite not observing dose‐dependent changes in echocardiographic indices of cardiac chamber size and systolic function. Further studies evaluating the potential clinical benefit of HD_pimo in dogs with MMVD are warranted.

Some might be skeptical of the value of evaluating the influence of SD_pimo and HD_pimo on renal and cardiac function in nonazotemic dogs with ACVIM stage B2 MMVD. However, it is well established that underlying renal dysfunction can exist before azotemia develops. Nonetheless, evaluating the dose‐dependent effect of pimobendan on cardiac and renal function in dogs with MMVD and refractory CHF (with or without azotemia) might bear more clinical relevance, as this cohort of dogs might have more potential to benefit from up‐titration of pimobendan. However, evaluating pimobendan in dogs with more advanced disease presents relevant challenges. Most notably, controlling for the effects of furosemide and angiotensin converting enzyme inhibitors or progressive disease on GFR. Our study sample mitigates these challenges and still provides valuable information in dogs with naturally occurring MMVD and cardiomegaly compared to healthy dogs or dogs with experientially‐induced MR. Interestingly, 7 dogs (23%) enrolled in our study had evidence of renal dysfunction based on GFR <2 mL/kg/min, despite all having creatine values ≤1.5 mg/dL. Based on the International Renal Interest Society staging these dogs had Stage I chronic kidney disease.

Additional limitations of this study should be acknowledged. This study only evaluated the effects of pimobendan over 7 to 10 days. The longer‐term effects of SD_pimo and HD_pimo on renal and cardiac function remain unknown. The severity of MR within ACVIM stage B2 is undoubtedly diverse and dogs with more severe MMVD might have had more to benefit from pimobendan. We elected not to specifically target these dogs because their selection would not have been based on consensus‐based guidelines and would have been somewhat arbitrary. Despite performing a power analysis based on an estimated treatment effect on GFR a priori, this study enrolled a small number of dogs. Type II error (false negative results) remains a possibility particularly for among group comparisons. This becomes evident when evaluating effect size relative to statistical significance. Recruiting more dogs or an alternative study design for example, cross‐over study, might have lowered the possibility of false negative results. However, cross‐over study designs present numerous additional challenges, particularly with client‐owned dogs, and recruiting more dogs would have increased study cost. Our assessment of renal function in this study does not represent the gold standard assessment of GFR in dogs. Renal clearance of inulin presents many challenges including assay availability, 24‐hour urine collection, and frequent urinary catheterizations.^32^ We opted to utilize plasma clearance of iohexol as detected by high‐performance liquid chromatography, which has been previously validated for measurement of GFR in dogs.^30^, ^32^, ^33^

In conclusion, our study was unable to demonstratable an effect of SD_pimo or HD_pimo on renal function in dogs with ACVIM stage B2 MMVD. High‐dose pimobendan (0.5‐0.6 mg/kg q12h) did not demonstrate advantages over SD_pimo in terms of cardiac function as assessed by selected echocardiographic indices of cardiac size and systolic function.

## CONFLICT OF INTEREST DECLARATION

Joshua A. Stern serves as Associate Editor for the Journal of Veterinary Internal Medicine. He was not involved in review of this manuscript. No other authors have a conflict of interest.

## OFF‐LABEL ANTIMICROBIAL DECLARATION

Authors declare no off‐label use of antimicrobials.

## INSTITUTIONAL ANIMAL CARE AND USE COMMITTEE (IACUC) OR OTHER APPROVAL DECLARATION

Approved by the IACUC at the University of California, Davis (protocol #20226).

## HUMAN ETHICS APPROVAL DECLARATION

Authors declare human ethics approval was not needed for this study.
